# Early Neurological Improvement Predicts Clinical Outcome After Thrombectomy for Distal Medium Vessel Occlusions

**DOI:** 10.3389/fneur.2022.809066

**Published:** 2022-03-07

**Authors:** Maud Wang, Yousra Farouki, Franny Hulscher, Benjamin Mine, Thomas Bonnet, Stephanie Elens, Juan Vazquez Suarez, Lise Jodaitis, Noémie Ligot, Gilles Naeije, Boris Lubicz, Adrien Guenego

**Affiliations:** ^1^Department of Interventional Neuroradiology, Erasme University Hospital, Brussels, Belgium; ^2^Department of Radiology, University Hospitals Leuven, Leuven, Belgium; ^3^Department of Neurology, Erasme University Hospital, Brussels, Belgium

**Keywords:** endovascular recanalization, distal thrombectomy, stroke, intravenous thrombolysis, prediction

## Abstract

**Background and Purpose:**

Good clinical outcome predictors have been established in mechanical thrombectomy (MT) for acute ischemic stroke (AIS) caused by large vessel occlusion (LVO). An early neurological improvement (ENI), defined as a reduction of ≥8 on the National Institutes of Health Stroke Scale (NIHSS), compared with the baseline score or an NIHSS of 0 or 1 at 24 h after MT, is a strong predictor of favorable outcome. We aimed to study the impact of ENI after MT for distal medium vessel occlusions (DMVO).

**Methods:**

We retrospectively analyzed the data of consecutive patients who underwent MT for a primary DMVO in one large academic center. We compared clinical outcomes between patients with DMVO stratified by ENI. Multivariate analyses were performed to determine the impact of ENI on good 90-day outcome (modified Rankin scale of 0–2) and identify factors contributing to ENI.

**Results:**

Between January 2018 and January 2021, 61 patients underwent an MT for an AIS with a primary DMVO. An ENI was seen in 24 (39%) patients (ENI+). Outcomes were significantly better in ENI+ patients, with 83% achieving a good outcome at 3 months vs. 43% for patients without ENI (ENI–; *p* = 0.019). ENI was an independent predictive factor of good clinical outcome even after adjusting for potential confounding factors [odds ratio 12.49 (1.49–105.01), *p* = 0.020]. The use of intravenous tissue plasminogen activator [IVtPA; Odds-ratio 6.59 (1.82–23.89), *p* = 0.004] was a positive predictor of ENI.

**Conclusion:**

ENI at day 1 following MT for DMVO stroke is a strong independent predictor of good to excellent 3-month clinical outcome.

## Introduction

It has been demonstrated that early neurological improvement (ENI) after mechanical thrombectomy (MT) or administration of Intravenous tissue Plasminogen Activator (IVtPA) for an anterior acute ischemic stroke (AIS) is a solid predictor of favorable long-term outcome (1–4). This is also applicable in late time windows (5) and for basilar artery occlusions (6).

In contrast, strokes due to distal, medium vessel occlusions (DMVO) have been understudied. Yet, recent advancements in retrieval devices and aspiration technology have allowed more and more centers to add thrombectomy to their therapeutic arsenal when treating DMVOs (7–11). While there are some data on predictors of a good clinical outcome (9, 12), the effect of ENI remains unstudied in this population.

Studies have already shown that significant reperfusion after MT is feasible for DMVOs in the M2-segment in a majority of cases along with an increase in functional independency at 3 months compared to the use of IVtPA alone (13).

Using the data from one large stroke center, we aimed to study the incidence and effect of ENI in patients with DMVO undergoing MT along with factors associated with ENI.

## Methods

This study was approved by our local institutional review board. Informed consent was waived due to minimal patient risk and practical inability to perform the study without the waiver.

The data that support the findings of this study are available from the corresponding author upon reasonable request. Adherence to the Strengthening the Reporting of Observational Studies in Epidemiology (STROBE) criteria (13) was enforced.

### Population

We retrospectively reviewed all patients admitted for AIS in one large stroke center between January 2018 and January 2021 and identified all those who underwent MT for a primary DMVO. The decision to perform the endovascular procedure was made by consensus on an individual patient basis.

We included patients older than 18 years who underwent MT for a primary DMVO, had a baseline imaging performed at our primary stroke center to ensure adequate assessment of the early infarct core volume, had a time from symptom onset to groin puncture of under 6 h, and underwent a secondary brain imaging study [fluid-attenuated inversion recovery (FLAIR) imaging on MRI] at day 3 after MT.

Thrombectomy procedures were carried out using standard of care recommendations. The patient's baseline clinical and radiological characteristics, procedure details, and outcomes were collected using standardized definitions.

Stroke etiologies were determined using the Trial of ORG 10172 in Acute Stroke Treatment (TOAST) criteria.

Before MT, baseline imaging assessed the infarct core volume (ml) [regional cerebral blood flow (rCBF) <30% on computed tomography perfusion (CTP)] using an automated software (RAPID, iSchemaView, Menlo Park).

Final infarct volume was assessed at day 3 on magnetic resonance axial FLAIR imaging using the Horos Dicom Viewer (Horos Project, Geneva, Switzerland) by the same board-certified neuroradiologist (A.G.) blinded to the initial CT, MT result, and clinical outcome. FLAIR axial sequences were performed on a 3T Siemens machine with the following parameters: repetition time (TR) 9,000 ms, time to echo (TE) 91 ms, slice thickness 4 mm, slice gap of 0.0, field-of-view (FOV) of 230 × 230 mm.

Infarct growth between the baseline CT and day 3 MRI was assessed as the difference between both infarct volumes.

### Clinical Definition

Early neurological improvement (ENI) was defined as already published: a reduction of 8 points or more on the National Institutes of Health Stroke Scale (NIHSS) or an NIHSS score of 0 or 1 at 24 h after MT (1, 2, 5, 6, 14, 15).

### Outcomes

The primary outcome evaluated was “good clinical outcome” at 3 months, defined as a modified Ranking Scale (mRS) score of 0–2, similar to recent randomized clinical trials. We compared patients with ENI (ENI+) and those without (ENI–) after MT for the primary and secondary clinical outcomes, including “excellent clinical outcome” (defined as a mRS of 0–1) and mortality rates at 3 months.

The following characteristics were compared between ENI+ and ENI– patients:

– Demographics, including age, gender, vascular risk factors, pre-stroke mRS
– Clinical presentation: initial NIHSS, heart rate, and blood pressure
– Stroke characteristics: imaging technique, use of IVtPA, time of onset (when known)
– Thrombectomy characteristics: type of anesthesia, devices/techniques used, number of passes, final reperfusion according to the final modified Thrombolysis in Cerebral Infarction (mTICI) scale (16) [mTICI 3, mTICI ≥2c (17), and mTICI ≥2b], and procedural complications (arterial perforation, arterial dissection, embolization in a new vascular territory, and vasospasm)
– Outcomes: hemorrhagic transformation (any type) and the rates of parenchymal hematomas [according to the European Cooperative Acute Stroke Study (18)] at day 1 of imaging)

### Statistical Analyses

Continuous variables are reported as mean (±SD) or median (interquartile range) and between-group comparisons were made with the Student's *t-*test or the Mann-Whitney *U-*test as appropriate according to normality testing. Categorical variables are reported as proportions and compared by χ^2^ or the Fisher exact test, as appropriate. The significance was set at *p* < 0.05 and was 2-sided. The normality of the variables' distributions was assessed by the Shapiro-Wilk test.

Univariate and multivariate binary logistic regression analyses were set to assess the association of ENI with the primary clinical outcome (mRS 0–2) after adjusting for potential baseline confounders known for their frequent association with clinical outcome or identified in univariate analysis (age, onset to recanalization delay, baseline core volume and NIHSS, occlusion side, IVtPA, or successful recanalization).

A second multivariate binary logistic regression assessed the potential predictors of ENI.

Results were expressed as odds ratio (ORs) with 95% confidence intervals (CIs). All statistical analyses were performed with the XLSTAT (XLSTAT 2020.1.3; Addinsoft; Paris; France).

## Results

**Figure 1:**
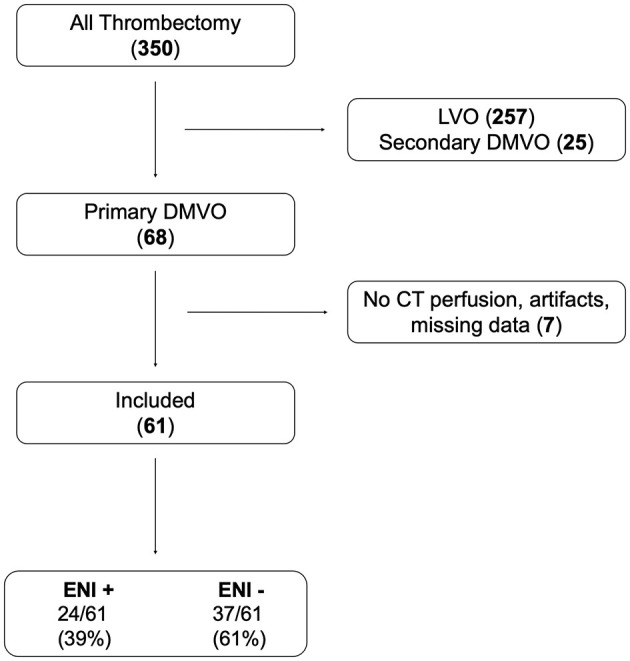
Flow chart.

A total of 61 patients were included (Figure 1), of which 24 (39%) showed ENI after MT.

There were no significant differences in terms of patient cardiovascular risk factors. Both groups had no significant discrepancy in baseline NIHSS [13 (10–18) for ENI+ vs. 11 (8–15) for ENI–, *p* = 0.105]. ENI+ patients showed a significantly smaller ischemic core volume on baseline imaging [0.3 ml (0.3–3.2) vs. 4 ml (0.3–8), *p* = 0.045] and a higher rate of left-sided occlusions (71 vs. 43%, *p* = 0.036). The patients had similar pre-stroke mRS (*p* = 0.643; Table 1).

**Table 1:** Baseline characteristics for patients stratified by early neurological improvement (ENI) status.

	**All**	**ENI–**	**ENI+**	***p*** **-value**
Number of patients	61 (100%)	37 (61%)	24 (39%)	
Age, years (median, IQR)	73 (64–82)	74 (68–81)	66 (60–83)	0.191
Female (%)	39 (64%)	25 (68%)	14 (58%)	0.589
Medical history				
High blood pressure (%)	54 (89%)	35 (95%)	19 (79%)	0.104
Diabetes (%)	11 (18%)	7 (19%)	4 (17%)	1.000
Hyperlipidemia (%)	36 (59%)	21 (57%)	15 (63%)	0.793
Weight (kg) (median, IQR)	75 (65–88)	75 (53–85)	75 (67–94)	0.367
Antiplatelets (%)	22 (36%)	16 (43%)	6 (25%)	0.174
Anticoagulants (%)	17 (28%)	9 (24%)	8 (33%)	0.383
Current smoking (%)	17 (28%)	9 (25%)	8 (33%)	0.564
Pre-stroke mRS of 0–1 (%)	57 (93%)	35 (95%)	22 (92%)	0.643
Clinical presentation				
Heart rate (bpm) (median, IQR)	75 (66–85)	72 (61–83)	75 (69–88)	0.182
Systolic blood pressure (mmHg) (median, IQR)	145 (131–163)	150 (131–170)	140 (131–157)	0.240
Diastolic blood pressure (mmHg) (median, IQR)	80 (75–88)	80 (75–85)	79 (74–91)	0.963
Baseline NIHSS (median, IQR)	12 (8–16)	11 (8–15)	13 (10–18)	0.105
IVtPA (%)	33 (54%)	15 (41%)	18 (75%)	**0.017**
Times				
Onset to puncture delay in min (median, IQR)	230 (170–277)	240 (210–312)	195 (165–240)	**0.045**
Unknown onset (%)	16 (26%)	9 (24%)	7 (29%)	0.771
Imaging				
Volume core, mL (median, IQR)	1.3 (0.3–8)	4 (0.3–8)	0.3 (0.3–3.2)	**0.045**
Volume TMax > 6 s (median, IQR)	40 (31–64)	40 (31–69)	38 (31–55)	0.716
Initial occlusion				0.601
A2 (%)	4 (7%)	2 (5%)	2 (8%)	
M2 (%)	35 (57%)	19 (51%)	16 (67%)	
M3 (%)	14 (23%)	10 (27%)	4 (17%)	
P2 (%)	8 (13%)	6 (16%)	2 (8%)	
Left side (%)	33 (54%)	16 (43%)	17 (71%)	**0.036**
Etiology				1.000
Atherosclerosis (%)	14 (23%)	9 (24%)	5 (21%)	
Cardio-embolic (%)	44 (72%)	26 (70%)	18 (75%)	
Unknown—Other (%)	3 (5%)	2 (5%)	1 (4%)	

*Significant p-values highlighted in bold*.

A larger portion of ENI+ patients received IVtPA (75 vs. 41%, *p* = 0.017) and had a shorter median delay between symptom onset and puncture [195 min (165–240) vs. 240 min (210–312), *p* = 0.045]. There were no significant differences concerning the anesthesia, MT technique, number of passes, reperfusion grade (mTICI ≥ 2b in 83% of ENI+ vs. 76% of ENI–, *p* = 0.746), or procedural complications.

Clinical outcomes at 90 days were significantly better in ENI+ patients, with 83% having a good outcome (mRS 0–2) vs. 43% of ENI– patients (*p* = 0.019) and 75% having an excellent outcome (mRS 0–1) vs. 16% of ENI– patients (*p* < 0.0001; Table 2).

**Table 2:** Procedural characteristics and clinical outcomes for patients stratified by ENI status.

	**All**	**ENI–**	**ENI+**	***p*** **-value**
Number of patients	61 (100%)	37 (61%)	24 (39%)	
Mechanical thrombectomy				
Admission mothership (%)	33 (54%)	16 (43%)	17 (71%)	0.085
Type of anesthesia				0.524
General anesthesia (%)	55 (90%)	32 (87%)	23 (96%)	
Conscious sedation (%)	4 (7%)	3 (8%)	1 (4%)	
CS then GA (%)	2 (3%)	2 (5%)	0 (0%)	
Technique				0.415
Contact aspiration (%)	20 (33%)	10 (27%)	10 (42%)	
Stent-retriever (%)	14 (23%)	8 (22%)	6 (25%)	
Combined (%)	27 (44%)	19 (51%)	8 (33%)	
Number of passes (median, IQR)	2 (1–3)	2 (1–3)	2 (1–3)	0.512
Procedural complication (%)	8 (13%)	7 (19%)	1 (4%)	0.132
mTICI ≥ 2b (%)	48 (79%)	28 (76%)	20 (83%)	0.746
mTICI ≥ 2c (%)	23 (38%)	12 (32%)	11 (46%)	0.419
Times				
Puncture to recanalization delay in min (median, IQR)	47 (25–70)	49 (27–74)	45 (24–61)	0.268
Onset to recanalization delay in min (median, IQR)	290 (225–330)	310 (242–330)	238 (194–314)	0.069
Early outcomes				
Day 1 NIHSS (median, IQR)	5 (2–10)	9 (6–15)	1 (1–4)	**<0.0001**
NIHSS shift (median, IQR)	−3 (−11 to 0)	−2 (−3 to 2)	−12 (−14 to −9)	**<0.0001**
Day 1 hemorrhagic transformation (%)	19 (31%)	12 (32%)	7 (29%)	1.000
ECASS PH-Type (%)	8 (13%)	4 (11%)	4 (17%)	0.533
Final stroke volume, mL (median, IQR)	16.3 (6.6–37.1)	19.6 (14–48.3)	8 (5–19.9)	**0.001**
Stroke progression, mL (median, IQR)	12 (5.6–27.9)	18 (8.6–37)	5.9 (4.2–13.3)	**0.001**
Long-term (3 months) outcomes				
mRS 0–1 (%)	24 (39%)	6 (16%)	18 (75%)	**<0.0001**
mRS 0–2 (%)	36 (59%)	16 (43%)	20 (83%)	**0.019**
Mortality (%)	5 (8%)	5 (14%)	0 (0%)	0.147

*Significant p-values highlighted in bold*.

Mortality rate was lower in ENI+ patients (0 vs. 14%), but this was not statistically significant (*p* = 0.147; Table 2).

Patients with ENI (ENI+) had a significantly smaller final stroke volume at day 3 [8 ml (5–19.9) vs. 19.6 ml (14–48.3), *p* = 0.001], paired with a more limited stroke progression (*p* = 0.001).

Early neurological improvement (ENI) was a predictive factor of good clinical outcome (mRS 0-2) in univariate analysis [OR 15.5 (4.34–55.30), *p* < 0.0001]. After adjusting for potential confounding factors, ENI was still an independent predictive factor of good clinical outcome (mRS 0–2) [OR 12.49 (1.49–105.01), *p* = 0.020].

A second multivariate analysis identified the use of IVtPA [OR 6.59 (1.82–23.89), *p* = 0.004] as a positive predictor of ENI.

## Discussion

Early neurological improvement (ENI) is a strong predictor of favorable clinical outcome in anterior-circulation strokes in both early (1–4, 14, 15, 19–21) and late time windows (5) and in basilar artery occlusions (6). Our study shows similar findings in patients with DMVO, suggesting that ENI is a strong independent predictor of improved clinical outcome with all conventional mRS dichotomizations.

The rate of ENI has been reported to occur in 28 (2) to 33% of patients after administration of IVtPA, 35% (20) after intra-arterial thrombolytic therapy, up to 43% (21) after MT for anterior circulation strokes within the 6 h window, and in 30% of basilar occlusions (6). We identified ENI in 39% of our cohort, which included a diverse array of patients. Hence, these “real world” findings reinforce the practical value of this early factor for prognostication and could be a useful tool when physicians are deciding on individual therapeutic courses and relaying information to the next of kin.

While our study suggests ENI to be a strong predictor of favorable outcome, a non-negligible percentage of patients without ENI also returned to functional independence at 90 days. Therefore, it would be counterproductive to systematically limit care in the absence of ENI after MT for DMVO stroke since this is not predictive of ineluctable major handicap or death.

We highlighted a smaller final stroke volume paired with a more limited stroke progression in ENI+ patients compared with patients without ENI. Limited infarct progression and final infarct volume by virtue of fast recanalization and tissue rescue may be one of the main factors explaining improved clinical outcomes in patients with DMVO (22). Furthermore, this association may help determine whether patients need to prioritize follow-up imaging at all, since patients with a limited stroke progression might be less prone to hemorrhagic transformation (23). This being said, no significant difference was highlighted in our study concerning this risk.

Interestingly, we identified the use of IVtPA as a significant predictor of ENI in patients undergoing MT for DMVO. This finding also needs to be substantiated by further studies but may suggest that physicians should not withhold intravenous thrombolytic therapy in such patients for fear of complications without careful consideration as the indication of MT in DMVO has not yet been demonstrated. Furthermore, one may question the safety of MT for DMVO in patients treated with IVtPA because of a potential risk of hemorrhagic complication. Given our results and despite the fact that this study was not focused on the use of IVtPA, physicians should not withhold endovascular management if they deem it to be the adequate management.

### Limitations

Our results suffer from inherent bias such as the retrospective nature of the data and the single-center design. We acknowledge that these results are not generalizable for now. These findings should be substantiated by prospective and multicenter studies for as long as no randomized trial has been published on DMVO. One should keep in mind that prospective studies may produce different results on this particular topic, and that despite many retrospective data in favor of DMVO MT, no randomized data is supporting its benefit yet.

Furthermore, we think our results could reinforce the practical value of this factor for early prognostication of patients after MT and help in determining which patients would need more intensive or earlier rehabilitation. Despite this, given the wide diversity of stroke care organizations, applicability of these findings needs to be evaluated for each specific center.

The mTICI score's use in DMVO strokes is debatable, and other recanalization scores may be developed as well.

## Conclusions

The presence of early neurologic improvement at day 1 post-MT for a primary DMVO stroke is a strong and independent predictor of good to excellent long-term clinical outcome despite relatively low baseline NIHSS. This is the first time ENI has been assessed and reported in a large population of patients with DMVO. Our recent findings could serve as a useful clinical tool for prognostication and therapeutic decision-making.

## Data Availability Statement

The data that support the findings of this study are available from the corresponding author upon reasonable request.

## Ethics Statement

The studies involving human participants were reviewed and approved by Erasme Hospital Ethical Committee. Written informed consent for participation was not required for this study in accordance with the national legislation and the institutional requirements.

## Author Contributions

AG conducted all the statistical analyses. All authors participated to the study design, data collection, data analysis, and writing of the manuscript. All authors contributed to the article and approved the submitted version.

## Conflict of Interest

The authors declare that the research was conducted in the absence of any commercial or financial relationships that could be construed as a potential conflict of interest.

## Publisher's Note

All claims expressed in this article are solely those of the authors and do not necessarily represent those of their affiliated organizations, or those of the publisher, the editors and the reviewers. Any product that may be evaluated in this article, or claim that may be made by its manufacturer, is not guaranteed or endorsed by the publisher.

## Abbreviations

AIS: Acute Ischemic Stroke
DMVO, Distal, Medium Vessel Occlusions, ECASS: European cooperative stroke study
ENI: Early Neurological Improvement
IVtPA: Intravenous tissue Plasminogen Activator
LVO: Large Vessel Occlusion
mRS: modified Rankin Scale
MT: Mechanical Thrombectomy
NIHSS: National Institute of Health Stroke Scale
mTICI: modified Thrombolysis in Cerebral Infarction.
